# Unilateral Pupil Sparing: Viral Oculomotor Neuritis With Concomitant Vestibular Neuritis

**DOI:** 10.7759/cureus.18979

**Published:** 2021-10-22

**Authors:** Elizabeth Mause, Mohammad Selim

**Affiliations:** 1 Internal Medicine, Creighton University School of Medicine, Omaha, USA

**Keywords:** oculomotor neuritis etiology, viral neuritis, cranial nerve three palsy, vestibular neuritis, dizziness, vertigo, ptosis

## Abstract

Unilateral oculomotor nerve palsy is a common clinical condition with various etiologies, including aneurysm, diabetes mellitus, central nervous system (CNS) infections, pituitary tumors, and ischemic changes. Due to the plethora of possible causes, early and thorough investigation is essential for treatment. We report the case of a 45-year-old male who presented with left ptosis, vertigo, and blurry vision and was diagnosed with oculomotor neuritis. Past medical history (PMH) was significant for hyperlipidemia, diabetes, and chronic kidney disease. Patient imaging revealed chronic left cerebellar infarction but no acute changes. Significantly, he was experiencing intractable nausea, dizziness, and vomiting attributed to concomitant vestibular neuritis. Infectious etiologies of oculomotor neuritis are rarely reported. However, idiopathic vestibular and facial palsies are commonly attributed to viral infection. The patient was treated with a steroid taper for viral vestibular neuritis, with noticeable clinical improvement to his oculomotor neuritis manifestations. This postulates a common viral etiology uniquely causing both oculomotor and vestibular neuritis.

## Introduction

The oculomotor nerve, cranial nerve three (CN III), has two main components: a general somatic efferent and a general visceral efferent. CN III is responsible for innervating four of the six extraocular muscles and the levator palpebrae superioris. It also innervates the constrictor pupillary and ciliary muscles of the eye that control pupillary constriction. Classically, a damaged somatic portion of the oculomotor nerve presents with a “down and out” eye, while a damaged visceral portion presents with a dilated pupil [1]. Pupil involvement is more likely to represent a critical underlying etiology, such as an aneurysm compressing the nerve, whereas a pupil sparing presentation is typically attributed to diabetic ophthalmoplegia. Viral etiology, although possible, has only been minimally reported in the literature. Vertigo is divided into peripheral and centrally caused categorizations. Peripheral vertigo is more common and associated with an inner ear etiology such as vestibular nerve infection. Vestibular neuritis presents with acute-onset nausea, vomiting, and imbalance, suspected to be due to viral-induced changes to the vestibular portion of cranial nerve eight (CN VIII) [2]. We present a unique case of unilateral oculomotor neuritis and concomitant vestibular neuritis in a middle-aged male.

## Case presentation

We present the case of a 45-year-old male patient with a past medical history of hypertension, hyperlipidemia, diabetes, and end-stage renal disease (ESRD). The patient presented to the emergency department (ED) with double vision, left eyelid drooping, left-sided headache, and vertigo associated with nausea, vomiting, and blurry vision. He also had retro-orbital pain and left ear itchiness. He never had symptoms like this before, and he denied any associated focal weakness, tingling, or numbness. He denied any history of cerebrovascular accident (CVA) but did have a prior episode of Bell’s palsy years ago. Notably, the patient was diagnosed with type 1 diabetes when he was 22. His diabetic disease course was complicated by proliferative diabetic retinopathy, vitreous hemorrhage, and end-stage kidney disease. He was diagnosed with ESRD two years prior to his presentation to the ED, with the placement of arteriovenous fistula and nightly peritoneal dialysis. At presentation, the patient's blood urea nitrogen (BUN) was 59 and creatinine was 13.8, consistent with his baseline BUN and creatinine ranges.

**Figure 1:**
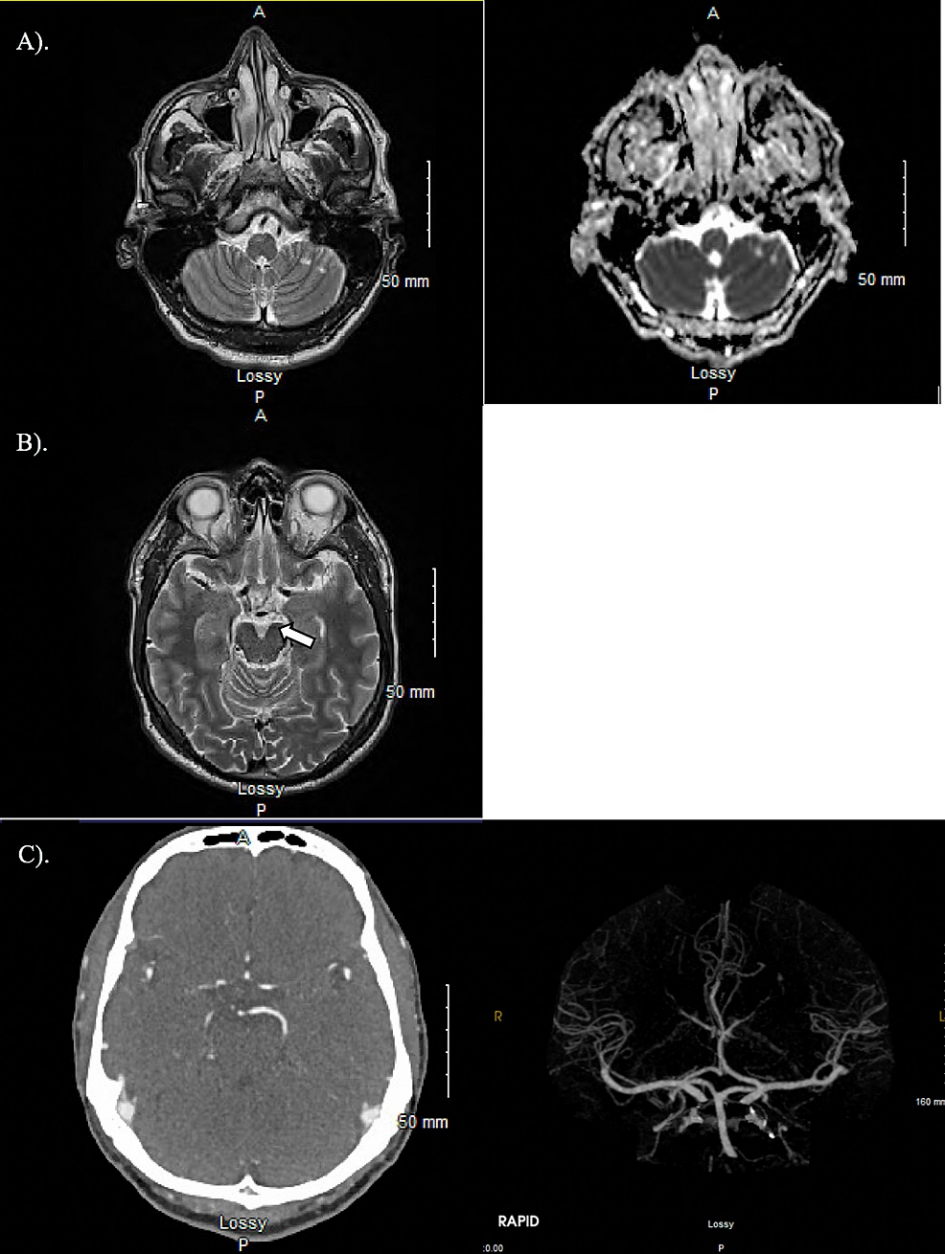
MRI imaging in a 45-year-old male with oculomotor nerve palsy and intractable nausea/vomiting A). Pre-contrast T2 axial MRI image with corresponding ADC diffusion sequence demonstrating two, small chronic left cerebellar infarct. B). Pre-contrast T2 axial MRI image demonstrating the absence of signal abnormality or compression left CN3 on imaging (see arrow) in a patient with left-sided ptosis. C). Computed tomography angiogram (CTA) head with and without contrast demonstrating normal-appearing posterior communicating arteries.

Physical exam in the ED revealed left eye ptosis and lateral squint with left eye adduction paralysis. Pupils were equal, round, and reactive to light. There was a positive left Dix-Hallpike maneuver. An intracranial process was excluded by computed tomography (CT) head, computed tomography angiography (CTA) head and neck, and magnetic resonance imaging (MRI). On imaging, no acute changes were demonstrated, but the MRI revealed two small, chronic left cerebellar infarctions - unlikely to be responsible for the current clinical presentation (Figure 1A).

The patient was admitted for further work-up. In-patient ophthalmology consult and ocular work-up confirmed weakness of left eye adduction and moderate left eye ptosis consistent with left pupil sparing third nerve palsy. The patient’s proliferative diabetic retinopathy was stable at that time. In-patient neurology consult provided additional support with confirmed exam findings of left eye ptosis with left eye adduction paralysis, associated right eye nystagmus on right lateral gaze, but impaired convergence, providing evidence against internuclear ophthalmoplegia. Imaging in the ED had excluded the possibility of an aneurysm so the other remaining common cause of oculomotor nerve palsy was uncontrolled diabetes. However, the patient’s hemoglobin A1C (HbA1C) had improved and average blood sugar readings were not elevated. HbA1C was measured at 6.6, which had improved from 7.3 one year ago. The patient was also consistent with follow-up for his diabetes and controlled his blood glucose well via insulin and diet (body mass index 22.9). Initially, there was hesitancy to start the patient on steroids due to his diabetes. Still, he was started on prednisone 60 mg on hospital day three for suspected concomitant vestibular neuritis due to intractable nausea and vomiting. His oculomotor and vestibular neuritis manifestations both improved gradually. He was discharged after four days total in the hospital with some residual nausea, but improved vomiting, dizziness, and double vision. These findings and clinical course were suggestive of a diagnosis of concomitant viral oculomotor and vestibular neuritis. He was discharged home with an eye patch on the left eye, a 10-day steroid taper course, meclizine, scopolamine patch, aspirin 81 mg daily, and increased daily atorvastatin from 20 mg to 40 mg.

## Discussion

Isolated oculomotor nerve palsy often presents with diplopia and ptosis with or without pupillary involvement. Intractable nausea and vomiting are not common symptoms. However, vestibular neuritis classically presents with nausea, vomiting, and vertigo. To our knowledge, this case represents the first report of simultaneous oculomotor and vestibular neuritis. This case also represents a rarely attributed cause of oculomotor neuritis - a viral infection. Notably, there has been a reported case of simultaneous oculomotor and facial nerve neuritis in a patient with systemic lupus erythematosus and Sjogren syndrome, and a reported case of oculomotor and optic nerve neuritis in a patient with herpes zoster ophthalmicus [3-4].

Isolated oculomotor cranial nerve palsy is most commonly associated with ischemic microvascular etiology. In a 2016 study analyzing hemoglobin A1C (HbA1C) levels related to cranial nerve three, four, and six palsies, the mean HbA1C associated with CN III palsy was 9.14 [5]. The recommended HbA1C goal for diabetic patients is below 7%, so our patient’s hemoglobin A1C level at 6.6 can be considered well-controlled [5]. His A1C level was lower than would be expected to induce palsies. Additionally, a typical ‘gloves and stocking’ distribution of sensory symptoms on a physical exam supports the diagnosis of diabetic peripheral neuropathy [1]. These sensory symptoms were absent in our patient.

Infectious causes of oculomotor neuritis, especially in cases of encephalitis or meningitis, are often mentioned as one of the underlying etiologies of oculomotor neuritis but rarely reported and not well-known by many clinicians. A few cases have been reported in patients with infectious mononucleosis and dengue fever [6-7]. Additionally, viral herpes zoster-induced CN III palsy has been reported in a patient who presented with herpes zoster ophthalmicus [4]. In one of the previously reported cases of viral oculomotor neuritis, MRI revealed abnormal enhancement and a lesion on CN III, clinically correlated to the patient’s symptoms [6]. However, a 2019 study found that out of 265 patients with cranial nerve three, four, or six palsies, only 60 had a corresponding nerve enhancement visualized with MRI [8]. CT, CTA, and MRI in our patient demonstrated no acute intracranial pathology (Figure 1B). With CN III palsy, it is also essential to rule out rare but potentially life-threatening posterior communicating artery aneurysms (Figure 1C).

Acute onset of nausea and vomiting are critical features in vestibular neuritis and were prominent in our patient’s presentation. Additionally, horizontal spontaneous nystagmus toward the unaffected ear is an essential sign of vestibular neuritis [9]. Our patient did have associated right eye nystagmus on right lateral gaze with suspected left ear vestibular neuritis. Notably, in a patient with vestibular neuritis, the Dix-Hallpike maneuver can accentuate the horizontal nystagmus - like in our patient who had a positive Dix-Hallpike maneuver. This requires careful physician differentiation via the patient's whole clinical picture (such as length and severity of dizzy episodes, presence of intractable nausea and vomiting) to exclude benign paroxysmal positional vertigo (BPPV) [10]. With right eye nystagmus and left eye adduction paralysis, it is also important to consider unilateral internuclear ophthalmoplegia. Left eye adduction paralysis with right eye nystagmus on rightward gaze is typical of a left medial longitudinal fasciculus (MLF) lesion and unilateral internuclear ophthalmoplegia. However, our patient had impaired convergence. Typical internuclear ophthalmoplegia includes the dissociation of convergence (i.e., convergence remains intact) and is an important differentiator of internuclear ophthalmoplegia from so-called pseudo internuclear ophthalmoplegia [11]. Pseudo internuclear ophthalmoplegia can present with myasthenia gravis or with CN3 palsy, as is the likely case in our patient. Additionally, many MLF brainstem lesions are visible on MRI imaging, which was normal in our patient. The patient’s history of Bell’s palsy also supports a diagnosis of concomitant viral oculomotor neuritis and vestibular neuritis. Vestibular neuritis is similar to Bell’s palsy in that it frequently results from a reactivated dormant herpes infection in Scarpa ganglia [12]. It is possible our patient has a dormant herpes infection that reactivated to cause his Bell's palsy in the past, and now to cause oculomotor and vestibular neuritis.

The patient was treated with steroids during his hospital stay and a 10-day taper at discharge. There is discord in the literature concerning the efficacy of steroids for vestibular neuritis therapy and far less discussion about its role in cases of viral oculomotor neuritis due to its rarity. Corticosteroids can improve the caloric test outcome in patients with vestibular neuritis but clinically evident recovery with steroids is limited [13]. However, prednisone during the first 10 to 20 days of the attack may shorten the illness [12]. Acyclovir has also been suggested for use in patients with herpes caused oculomotor neuritis [4]. Clinically, our patient’s symptoms improved after starting steroids. His vomiting and double vision were improved upon discharge.

## Conclusions

Oculomotor neuritis is a common clinical phenomenon usually attributed to vascular aneurysm compression or as a sequela of diabetes. Viral etiology is a recognized possibility for oculomotor neuritis but has only been minimally identified in the current literature. Oculomotor neuritis has been noted to present with concomitant facial nerve or optic nerve involvement but has not been reported with only accompanying vestibulocochlear nerve involvement. Due to limited cases of viral oculomotor neuritis in the literature, the efficacy of steroids has not been analyzed. In vestibular neuritis, steroid benefit is controversial but may have some advantages.
